# Lateral extension and attachment of mesh to the lateral vagina during laparoscopic sacrocolpopexy: a modified technique aimed at lowering recurrences in the anterior vaginal compartment. A surgical video

**DOI:** 10.1007/s00192-022-05338-8

**Published:** 2022-09-09

**Authors:** E. Bousouni, D. Sarlos

**Affiliations:** Department of Gynecology and Gynecological Oncology, Kantonsspital Aarau, Aarau, Switzerland

**Keywords:** Laparoscopic sacrocolpopexy, Laparoscopic prolapse surgery, Minimal invasive prolapse surgery

## Abstract

**Purpose/objective:**

Laparoscopic sacrocolpopexy has been demonstrated to be the gold standard of prolapse surgery in cases with apical defect. Most recurrences seem to occur in the anterior compartment, especially if a paravaginal defect is present. To reduce the incidence of anterior recurrence after laparoscopic sacrocolpopexy we modified our previous published technique by placing the anterior mesh not only deep under the bladder but also laterally and fixing it to the lateral edge of the vagina. With this video article, we would like to show and explain our modified technique and demonstrate how lateral mesh placement can be easily and safely performed using laparoscopy.

**Methods:**

The video demonstrates our modified technique with lateral extension and fixation of the anterior mesh to the lateral vagina during laparoscopic sacrocolpopexy in a patient with severe uterine prolapse (grade III) and a large cystocele (grade III). Special emphasis is given to the topographical anatomy of the paravaginal space and the surgical technique of lateral fixation.

**Results:**

This modified new technique shows excellent perioperative results in more than 100 cases without any occurrences of lesions of the ureters. Our initial experience also shows very good anatomical results in all three compartments.

**Conclusions:**

Paravaginal dissection and exposure of the ureters to extend the mesh placement and fixation to the lateral border of the vagina in the anterior compartment during laparoscopic sacrocolpopexy seem to be feasible and safe, helping to significantly reduce the risk of anterior recurrences. Prospective data are needed to evaluate this interesting technique.

**Electronic supplementary material:**

The online version of this article (10.1007/s00192-022-05338-8) contains supplementary material. This video is also available to watch on http://link.springer.com/. Please search for this article by the article title or DOI number, and on the article page click on ‘Supplementary Material’

## Aim of the video/introduction

This is a teaching video to show how lateral dissection of the paravaginal space during laparoscopic sacrocolpopexy can be achieved easily, as many urogynaecologists may not be familiar with the topographic anatomy of the distal ureter. The video demonstrates our modified technique with exposure of the paravaginal part of the ureter in order to extend anterior mesh placement and fixation to the lateral edge of the vagina. Our technique is not a paravaginal repair technique in which the lateral border of the distal and mid-vagina would be reattached to the arcus tendineus fascia pelvis, but we are extending the apical support down to the mid-vagina. In our opinion this could be an important step in avoiding lateral anterior recurrences after laparoscopic sacrocolpopexy.

Laparoscopic sacrocolpopexy is the gold standard in the treatment of apical prolapse [1, 2]. Prolapse recurs in the anterior compartment in up to 10% of cases, depending on the follow-up time [3]. Anterior recurrence after sacrocolpopexy is difficult to treat, especially in cases where the apex is still well suspended and the patient is sexually active. Hence, we aimed to adapt our initially published technique [4] to avoid anterior recurrence.

The recurrence rate in the anterior compartment in our group was 6% and we have seen that this is particularly common in patients with initial paravaginal defects [5]. With this new technique the lateral part of the vagina is much more effectively suspended and could lead to fewer anterior recurrences.

## Method

The video shows a 54-year-old female patient (PII/GIII) with no family history of prolapse and no prior surgery for pelvic organ prolapse, with a grade III uterine prolapse and a grade III cystocele (according to the International Continence Society/International Urogynecology Association classification), and a pronounced paravaginal defect. The hysterectomy is performed supracervically to prevent lateral vaginal erosion. The self-designed manipulator type SCHÄR [6] is inserted into the cervical stump, to have optimal manipulation of the vagina. Dissection starts at the level of the promontory, where the peritoneum is opened and the autonomic nerve fibres of the superior hypogastric plexus are dissected and pushed to the left side [4, 7]. The crossing fibres of the superior hypogastric plexus are then pushed dorsally before the peritoneum is incised. The rectovaginal space is then opened and the rectum is gradually detached from the posterior wall of the vagina. The perirectal fat should be positioned dorsally, so that an atraumatic preparation can be performed in a few steps in non-operated patients until the muscle levator ani can be visualised with no problems.

**Fig. 1:**
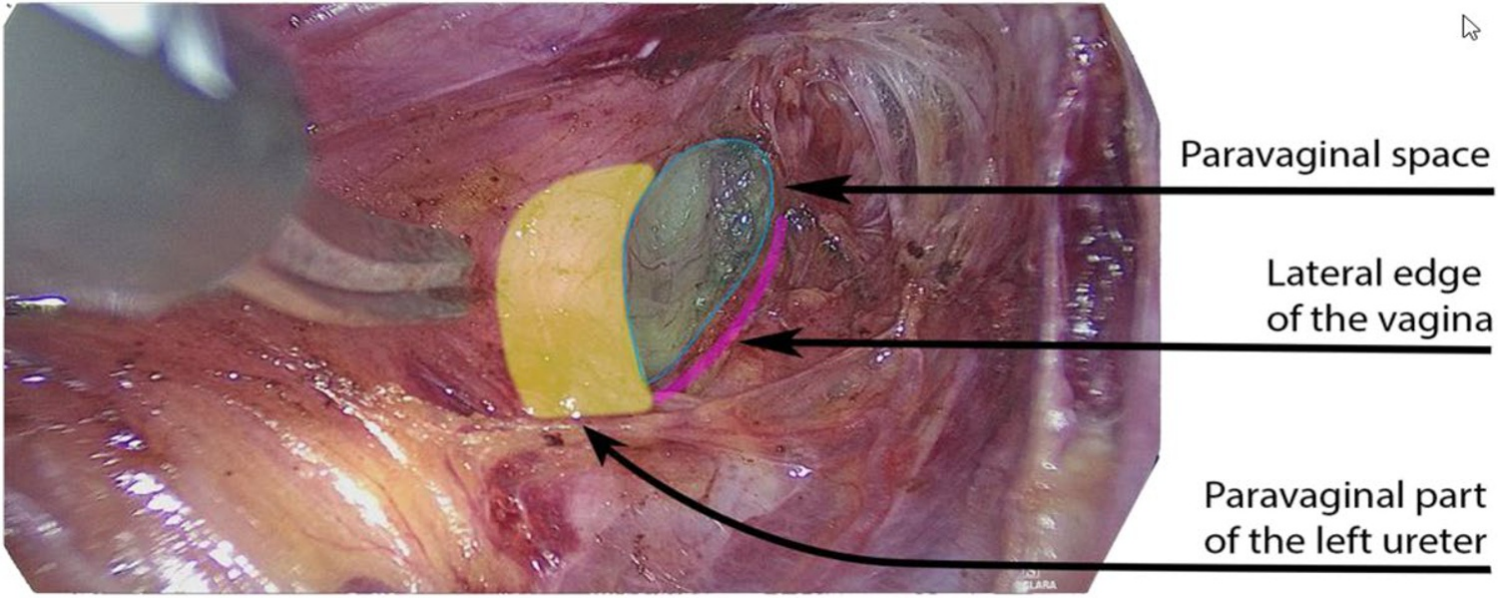
Paravaginal exposure of the ureter. Once it is identified, the paravaginal space can be opened easily

In the anterior compartment, the bladder is lifted and then the paravaginal space is opened. For patients who have not been operated on before, this step can usually be done by atraumatic dissection. First, we open the vesico-vaginal space and separate the bladder from the vagina to the level of the bladder trigone. Lateral dissection is performed by opening the paracolpic space and exposing the lateral edge of the vagina. Using the vaginal and cervical stump retractor, the lateral edge of the vagina can easily be identified and the paracolpic space is opened. The ureter is then identified at about 2–3 cm before entering the bladder wall and pushed laterally to avoid lateral injury. The paracolpic veins should remain on the side of the vagina to avoid bleeding. In the median plane, the bladder is dissected up to the level of the trigone. The anatomy of this step of the dissection is illustrated in Fig. 1 for better understanding. The anterior mesh is than sutured to the distal vagina in the midline and laterally to the edge of the vagina with non-absorbable sutures.

**Fig. 2:**
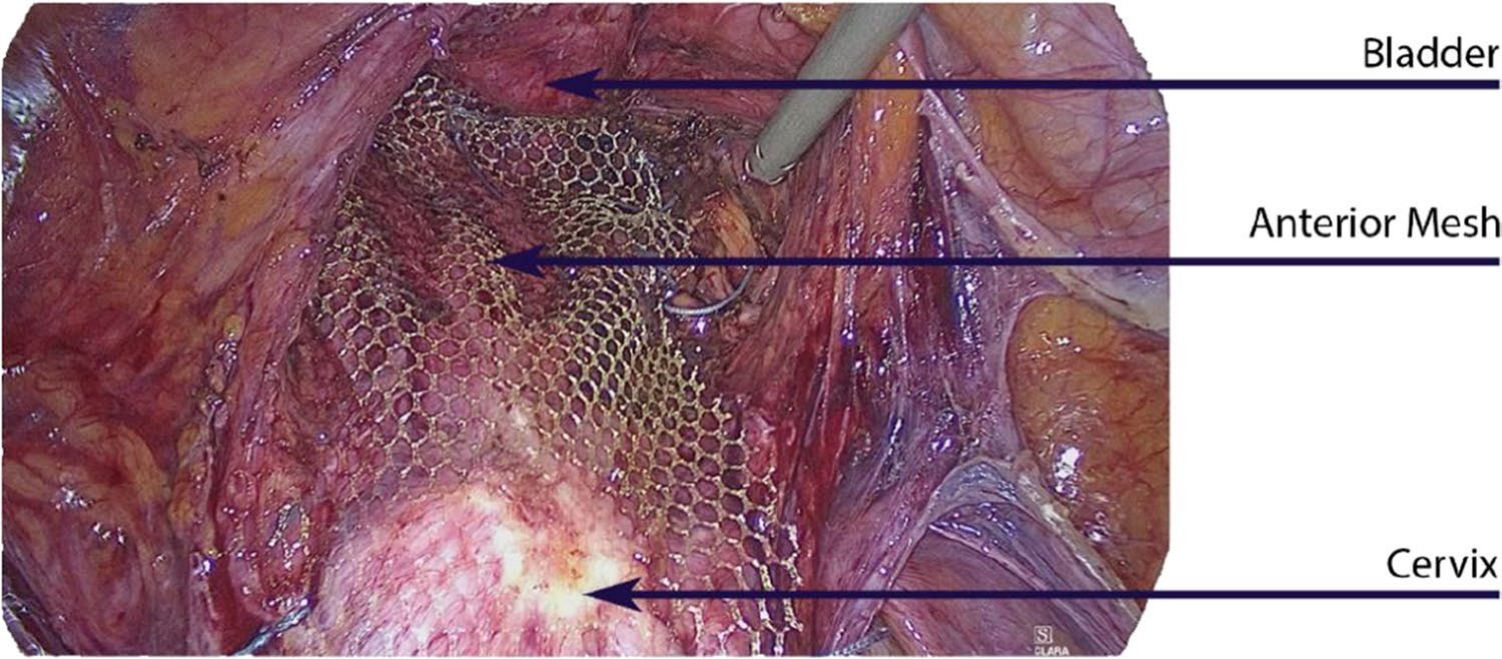
Suture of the mesh, deep paravaginal I). Once it is identified, the paravaginal space can be opened easily

**Fig. 3:**
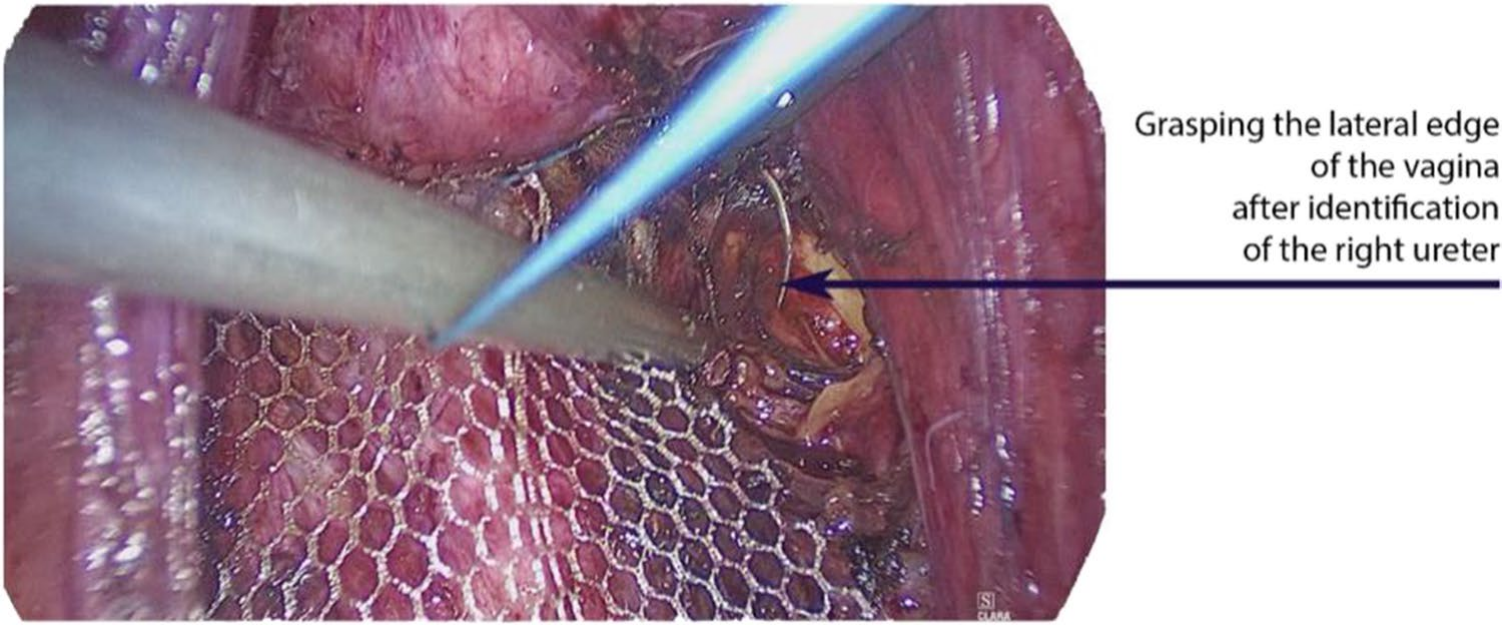
Suture of the mesh, deep paravaginal II. The paravaginal suture is performed on the right side by grasping the lateral part of the vagina completely and securing it together with the mesh. This not only stretches the vagina but also suspends the lateral parts of the vagina, which in our opinion significantly reduces the risk of lateral recurrence

The reason for the extensive paracolpic dissection is to place the anterior mesh on the full width of the vagina and fix it at the lateral edge of the vagina to guarantee optimal elevation of the lateral part of the vagina during laparoscopic sacrocolpopexy (Figs. 2, 3). It should be noted that without preparation of the ureter these sutures should not be performed, as there would be a substantial risk of a ureteral lesion or stricture.

We use the PelviGYNious Mesh®, a macroporous polypropylene, which is extremely suitable. All sutures are made using the extracorporeal knotting technique with Ethibond 2–0, non-absorbable suture. The ventral mesh is fixed with two lateral stitches on each side and one in the midline beyond the trigone of the bladder. On the dorsal side the second mesh is attached to the ventro-lateral part of the levator ani muscle and proximally to the sacro-uterine ligaments.

**Fig. 4:**
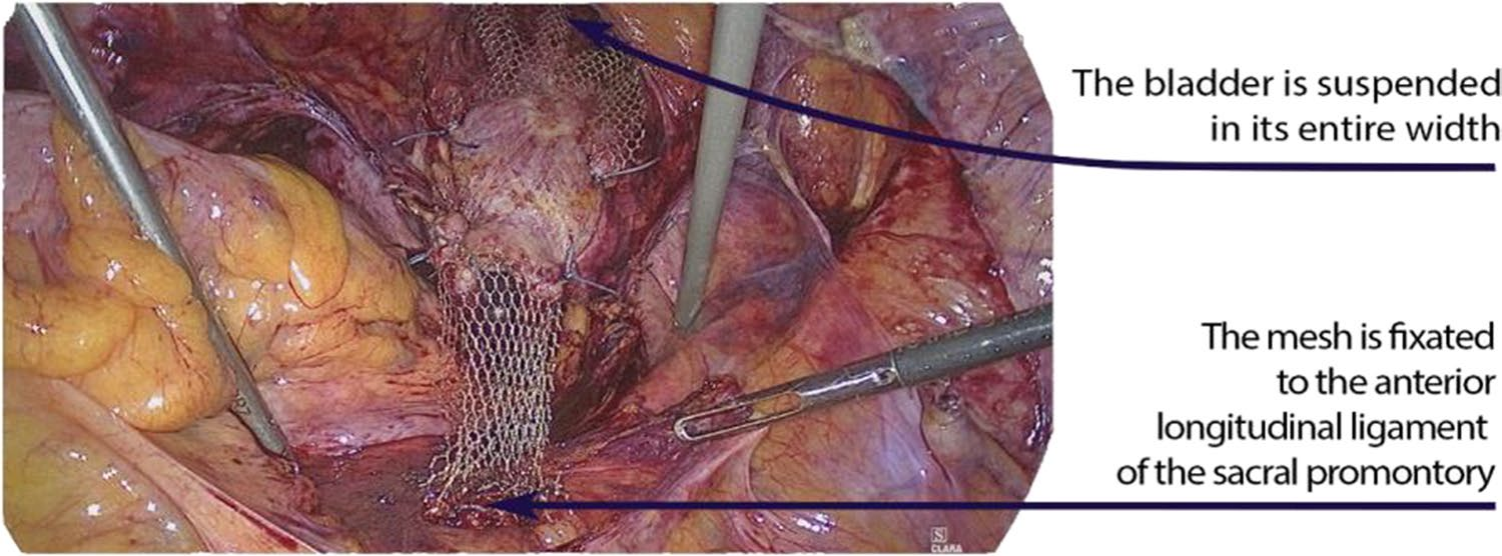
The condition after attachment. The ventral mesh can be seen, which is also attached laterally, thus suspending the bladder for its entire width

Anterior and posterior mesh are cut to the appropriate size so that a more or less tension-free attachment is possible. The ventral and dorsal mesh is attached to the longitudinal ligament with laparoscopic suturing with Ethibond 2–0 sutures. Technically, this is easier to do with the left hand owing to the angle of the instruments; the median sacral artery should be exposed and protected here to avoid bleeding. One should also be aware and watch for deep iliac veins on the left side. An adequate portion of the ligament should be taken to ensure optimal fixation. Usually, we place two sutures at the promontory. Figure 4 shows the final view after fixing the anterior and posterior mesh on the promontory.

Finally, a complete peritonealisation of the mesh is very important to avoid intestinal incarceration. We usually use a continuous Vicryl 2–0 suture.

## Results

Perioperative results of laparoscopic sacrocolpopexy with lateral extension of mesh placement and fixation to the lateral vagina are excellent. We have performed surgery in more than 100 patients in the last 2 years using this technique. Intraoperative complications are very rare, especially because we did not see ureteral lesions or strictures.

As we follow all our patients after laparoscopic sacrocolpopexy, we can report a significant improvement in anatomical outcome in the anterior compartment, at least in short-term follow-up.

## Conclusion

This adapted technique of the lateral extension of anterior mesh placement and fixation to the lateral part of the vagina leads to optimal suspension of the full width of the vagina during laparoscopic sacrocolpopexy. It is not a paravaginal repair technique but extends the apical support to the mid-vagina. The technique is easily feasible and safe and could lead to less lateral recurrences in the anterior compartment after laparoscopic sacrocolpopexy.

Further studies in the form of randomised controlled trials with large patient cohorts are needed to support these promising preliminary results.

## Supplementary information

ESM 1 (MP4 154655 kb)
